# Supplementary material for: Large-scale discovery of protein interactions at residue resolution using co-evolution calculated from genomic sequences
Source: Nat Commun. 2021 Mar 2;12:1396. doi: 10.1038/s41467-021-21636-z (PMC7925567; doi:10.1038/s41467-021-21636-z)
Supplement: Supplementary file 12 — Supplementary Data 10 [file 41467_2021_21636_MOESM12_ESM.zip › supplementary/allpdb0046_CYB_QCR8/allpdb0046_CYB_QCR8.pdf]

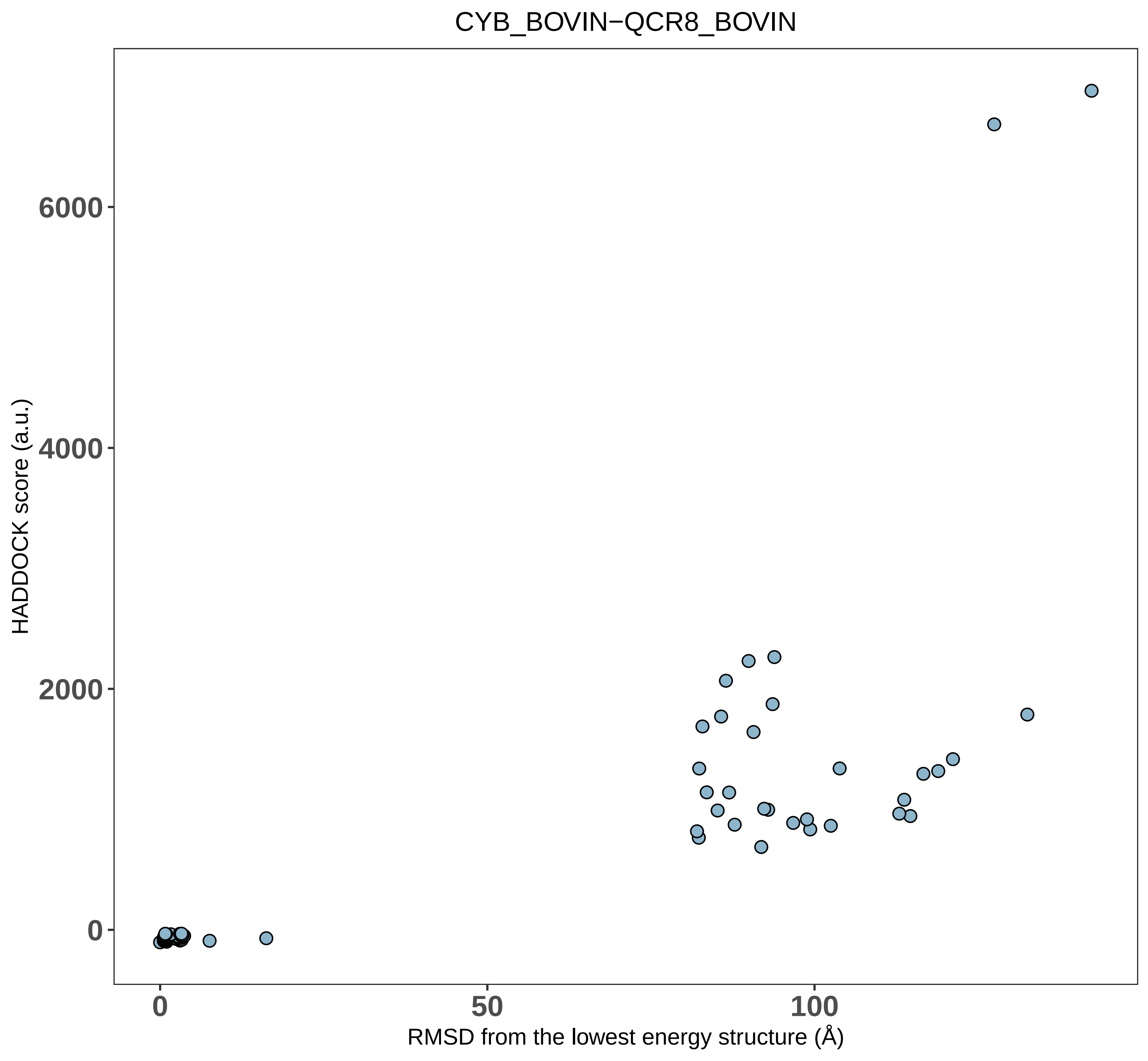
CYB_BOVIN−QCR8_BOVIN
HADDOCK score (a.u.)
6000
4000
2000
0
0
50
100
RMSD from the lowest energy structure (Å)
